# Insulin-like growth factor-1 signaling in renal cell carcinoma

**DOI:** 10.1186/s12885-016-2437-4

**Published:** 2016-07-12

**Authors:** Adam F. Tracz, Cezary Szczylik, Camillo Porta, Anna M. Czarnecka

**Affiliations:** Department of Oncology with Laboratory of Molecular Oncology, Military Institute of Medicine, Szaserow 128, 04-141 Warsaw, Poland; First Faculty of Medicine, Medical University of Warsaw, Warsaw, Poland; Department of Medical Oncology, IRCCS San Matteo University Hospital Foundation, Pavia, Italy

**Keywords:** Renal cell carcinoma (RCC, ccRCC), Insulin-like growth factor-1 (IGF-1), IGF-1 receptor (IGF1R)

## Abstract

Renal cell carcinoma (RCC) incidence is highest in highly developed countries and it is the seventh most common neoplasm diagnosed. RCC management include nephrectomy and targeted therapies. Type 1 insulin-like growth factor (IGF-1) pathway plays an important role in cell proliferation and apoptosis resistance. IGF-1 and insulin share overlapping downstream signaling pathways in normal and cancer cells. IGF-1 receptor (IGF1R) stimulation may promote malignant transformation promoting cell proliferation, dedifferentiation and inhibiting apoptosis. Clear cell renal cell carcinoma (ccRCC) patients with IGF1R overexpression have 70 % increased risk of death compared to patients who had tumors without IGF1R expression. IGF1R signaling deregulation may results in *p53, WT, BRCA1, VHL* loss of function. RCC cells with high expression of IGF1R are more resistant to chemotherapy than cells with low expression. Silencing of IGF1R increase the chemosensitivity of ccRCC cells and the effect is greater in *VHL* mutated cells. Understanding the role of IGF-1 signaling pathway in RCC may result in development of new targeted therapeutic interventions. First preclinical attempts with anti-IGF-1R monoclonal antibodies or fragment antigen-binding (Fab) fragments alone or in combination with an mTOR inhibitor were shown to inhibit in vitro growth and reduced the number of colonies formed by of RCC cells.

## Background

Renal cell carcinoma (RCC) comprise 2–3 % of malignant tumors in adults. Clear cell RCC (ccRCC) develops from epithelium of the proximal tubules and is the most common a histological type of RCC – diagnosed in 60-70 % of cases. Initial treatment of RCC is most often partial or radical nephrectomy. Nevertheless one third of patients are diagnosed with synchronous metastases [1]. IGF-1 plays an important role in protection from apoptosis and regulation of cell growth. Deregulation in downstream IGF-1 receptor results in angiogenesis, cell viability, proliferation and invasion. IGF1R expression is poor prognostic factor especially among those presenting with high-grade disease at the initial evaluation. RCC cells with high expression of IGF1R are more resistant to chemotherapy than cells with low expression of that receptor [2]. IGF-1 and insulin share overlapping downstream pathways of cancer cell metabolism. Cell line based studies have shown that down-regulation, knock-out, pharmacological inhibition of IGF-IR may in turn increase in IR signaling and therefore insulin analogs may promote cancerogenesis [3]. On the other hand first preclinical attempts with anti-IGF-1R monoclonal antibodies or fragment antigen-binding (Fab) fragments alone or in combination with an mTOR inhibitor were shown to inhibit in vitro growth and reduced the number of colonies formed by of RCC cells [4, 5]. All these findings suggest that IGF1R expression is significant in ccRCC and understanding of the molecular mechanism of IGF-1 and insulin signaling pathway in RCC may give opportunity to design molecular markers of disease or even finding a new molecular targets for drugs.

### Renal cell carcinoma

Renal cell carcinoma is the seventh most common malignancy with expected 5-year survival rate of 72 %. About 64 % of patients are diagnosed with localized disease [6]. RCC has highest incidence in highly developed countries. RCC includes several types of cancer: clear cell RCC, papillary RCC, chromophobe RCC and also rare cancer like, urothelial carcinoma, collecting duct carcinoma and renal medullary carcinoma [7]. The most common type is clear cell RCC that is diagnosed in up to 70 % of cases. Among those up to 60-90 % of sporadic cases of ccRCC exhibit VHL gene loss, silencing, mutation or promoter hypermethylation. The VHL protein (pVHL) is an E3 ubiquitin ligase of hypoxia inducible factor (HIF). It targets HIF for degradation by the proteasome. In the absence of functional pVHL HIF is accumulated in the nucleus where it acts as a transcription factor for vascular endothelial growth factor (VEGF), platelet derived growth factor (PDGF), multidrug resistance pump (MDR-1), cyclin D1 (CCND1), ENOLASE2 (ENO2), Egl-9 Family Hypoxia-Inducible Factor 3/HIF Prolyl Hydroxylase 3 (EGLN3), angiopoietin-like 4 (ANGPTL4), glucose transporter 1 (GLUT1), insulin-like growth factor-binding protein 3 (IGFBP3), and erythropoietin (EPO) [8, 9]. Management of RCC is usually initiated with partial or radical nephrectomy. Ablation procedure is alternative for small renal masses in patients who are ineligible for surgery [10, 11]. Adjuvant therapy has no proven efficacy on management of RCC. Only systemic treatment of metastatic disease has been shown as effective in phase III trials [1, 7]. There are 3 major groups of systematic treatment that are used for metastatic RCC: cytokines, mTOR inhibitors and anti- vascular endothelial growth factor (VEGF) - targeted drugs [7]. Cytokine based immunotherapies included interferon-alpha [12] and high dose interleukin 2 (IL-2) [13], while mTOR inhibitors approved for RCC treatment are everolimus [14] and temsirolimus [15]. VEGF pathway inhibitors used are 1) tyrosine kinase inhibitors as sorafenib [16], sunitinib [17], pazopanib [18], and axitinib [19], and 2) anti-VEGF monoclonal antibody – bevacizumab [20] (Table 1). New drugs that modulate immunological response including anti-CTLA-4 (cytotoxic T lymphocyte antigen 4) antibody and anti-PD-1/PDL-1 (programmed cell death 1) antibody have been developed recently [7, 21, 22]. Anti-CTLA-4 or anti-PD-1 antibodies inhibit the immunosuppression between T cells and APC (antigen presenting cells) including cancer cells which stimulate anti-tumor response [21, 22]. Most recently nivolumab - anti-PD-1 monoclonal antibody - was shown as effective against RCC in trail using everolimus as comparator [23].

**Table 1:** Drugs used in RCC therapy

Group of drug	Drugs	Ref.
cytokines	1) Interleukin 2 - overall response rate - 15 %, complete response rate - 5 %, achieved by high dose. Problems with selection of patients who may benefit from treatment. 2) Interferon alfa - inferior to most new agents considering PFS (progression free survival), except in combination with bevacizumab.	[13, 100]
VEGF-targeted drugs	1) Sorafenib – second and subsequent lines of treatment. 2) Sunitinib – first line treatment for metastatic RCC. 3) Pazopanib – non-inferior to sunitinib 4) Axitinib – treatment refractory RCC. 5) Bevacizumab – used with interferon alfa. Superior PFS when compared with monotherapy of interferon alfa.	[7, 101, 102]
mTOR inhibitors	1) Temsirolimus – for patient with poor risk as a first line drug^a^ 2) Everolimus – used as a second line or third line drug.	[7, 14, 17]

^a^the five Memorial Sloan Kettering Cancer Center (MSKCC) factors plus metastasis in more than one organ

### General IGF-1 function

Insulin-like growth factor 1 (IGF-1, somatomedin C) is a natural anabolic peptide hormone produced mainly by hepatocytes. IGF-1 with molecular weight of 7649 Da is built by 70 amino acids and single polypeptide chain with three intramolecular disulfide bridges. Production of IGF-1 is stimulated by growth hormone (GH) secreted by anterior pituitary. IGF-1 production is also stimulated by insulin and has influenced on reduction of lipolysis, glycolysis, inhibition of lipolytic function of adrenaline, embryonic growth and differentiation of cells. IGF-1 may also be released independently of GH. Circulating IGF-1 produced in liver acts in endocrine manner, but locally produced IGF-1 acts also in an autocrine manner. IGF functions therefore both as circulating hormone and tissue growth factor. Circulating IGF-1 forms a complex with two other proteins – the IGF binding protein (IGFBP) and the acid labile subunit (ALS). Six different IGFBPs were characterized, but about 75 % of serum IGFs are bound to IGFBP3 and only 1 % of serum IGF-1 is free-bioactive form [24]. IGFBPs are also mostly synthesized in the liver. Nevertheless IGFs and IGFBPs are also produced in other organs, acting locally in autocrine and paracrine manner and mediating stromal - epithelial cell interactions [25]. IGFBPs acts in a competing manner against IGFR (IGF receptors) and IGFBP proteases. IGF-1 and IGFBP-3 complex play crucial role in mitogenesis, cell differentiation and survival [26]. *IGF-1* null mice die shortly after birth [27].

### Circulation of IGF-1

High level concentrations of circulating IGF-1 are related with higher risk of prostate, colorectal and breast cancers [28–30]. Circulating concentrations of IGFBP-3 is associated with increased risks of breast cancers in postmenopausal women and prostate cancer in men [28, 29, 31]. Transgenic mouse with deletion in liver-specific *IGF-1* that result 75 % reduction in circulating IGF-1 exhibit reduction in development of colon cancer and reduced growth tumor xenografts [31, 32]. Laron syndrome is genetic condition characterized by GH insensitivity and in consequence IGF-1 deficiency [33]. People with Laron syndrome are resistant to cancer what was shown by Steuerman et al. [34]. They found that none of the 230 patients with Laron syndrome developed cancer and that only 1 out of 116 patients with inborn IGF-1 loss was diagnosed with malignancy [34].

### IGF-1 receptor and insulin receptor homology

**Fig. 1:**
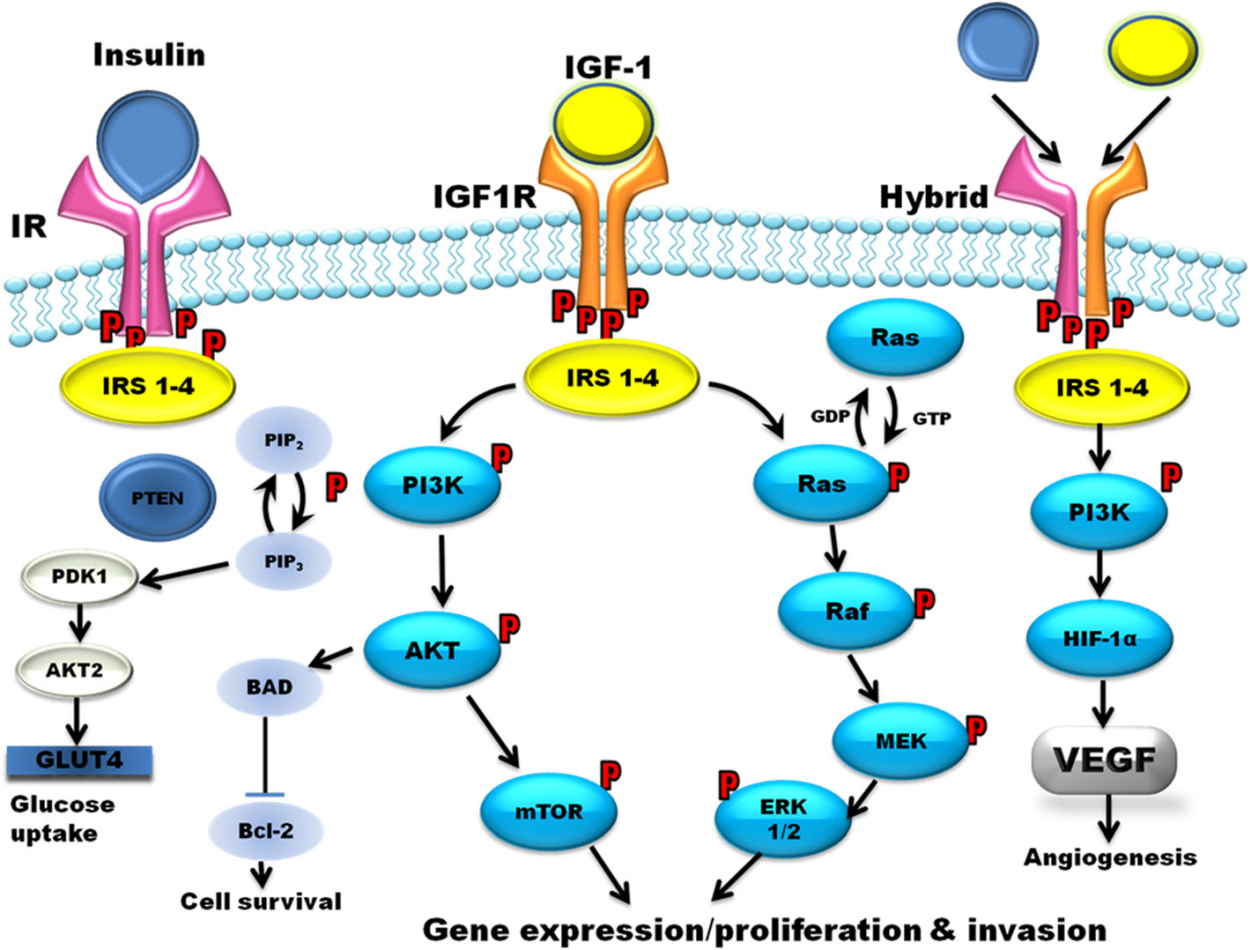
Schematic representation of downstream signaling of IGF1R. AKT, protein kinase B; AMPK, AMP-activated protein kinase; Bcl-2, B-cell lymphoma 2; BAD, B-cell CLL/lymphoma 2 antagonist of cell death; ERK 1/2, extracellular-signal-regulated kinase 1/2, IGF1R, insulin-like growth factor 1 receptor; IR, insulin receptor; IRS1-4, insulin-like receptor substrate 1–4; MEK, mitogen-activated protein kinase kinase; mTOR, mammalian target of rapamycin; PI3K/AKT, phosphatidylinositol 3-kinase/AKT; PDK1, 3-phosphoinositide-dependent protein kinase; PIP2, phosphatidylinositol 4,5-bisphosphate; PIP3, phosphatidylinositol 3,4,5-trisphosphate; PTEN, phosphatase and tensin homolog; GLUT4, Glucose transporter type 4; HIF-1α, Hypoxia-inducible factor 1-alpha, VEGF, Vascular endothelial growth factor

IGFR-1 is a transmembrane receptor with tyrosine kinase activity and is built of two α-subunits (located extracellularly) and two β-subunits (spanning the membrane and activating intracellular signal transduction). Both the α and β subunits are synthesized from a single precursor mRNA. IGF1R shares a high structural homology with the insulin receptor (IR) – has more than 50 % in the overall amino acid sequence and in particular 84 % similarity in the tyrosine kinase domain and 45–65 % in the ligand-binding domain. Moreover ligand-dependent activation of the IGF1R and IR activates almost identical downstream signaling pathways [35]. After IGF-1 binging activation of tyrosine kinase (β-subunits) results in downstream signaling via IR substrate proteins (IRS1-4), Src homology 2 domain containing transforming protein 1 (Shc), GRB2-associated binding protein 1 (Gab-1), Casitas B-lineage Lymphoma proto-oncogene E3 ubiquitin protein ligase (Cbl), Phosphatidyl Inositol 3-Kinase (PIK3), Protein kinase B (Akt), mammalian target of rapamycin (mTOR), mitogen-activated protein kinase (MAPK) and signal regulatory protein family [36]. Insulin and IGFs have a great homology and can have cross-reactivity upon receptors. Moreover hybrid receptors - constituted of IR and IGF1R heterodimers – have been shown to have cellular biological effects resembling those of the IGF1R and were found in colon cancer, thyroid cancer and breast cancer cell lines and tissues [37]. To complicate the interaction even more there are two IR isoforms, arising in the cell by alternative splicing of exon 11 – isoform IR-A, that lacks exon 11, and isoform IR-B – containing exon 11. Insulin does not bind to the hybrid receptors, but binds to IR-A, IR-B, and IGF-1R but binds to the IGF-1R with much lower affinity than to the IR. IGF-I binds to the IGF-1R, hybrid receptors, and IR but has much lower affinity for the IR than IGF-1R [3]. In total insulin and IGF-1 interact with six receptors: the type I IGF receptor (IGF1R), the IRA (IR-A, predominantly expressed in fetal tissue), the IRB (IR-B, predominantly expressed in adult tissue), hybrid receptors of IGF and IR-A, hybrid receptors of IGF and IR-B, and hybrid receptors of IR-A and IR-B [38, 39]. Insulin and IGF-1 while binding to IGF1R, IR-A, IGF1R/IR-A, mediate mostly mitogenic signaling (Ras > MEK > Erk1/2 pathway), while binding to IR-B activate mostly metabolic pathway (PI3K > Akt > mTOR) [24, 36, 40]. As a result both insulin and IGF-1 can act through the hybrid receptors and through the specific receptor for their ligand (Fig. 1). Activation of all receptors (IR, IGF1R, hybrid) which are tyrosine kinase cell-surface receptor result in phosphorylation of IR substrate proteins (IRS 1–4). It activates two key signal-transduction pathways. The GTPase Ras-Raf-MEK-ERK1/2 pathway activates gene expression that result in cells proliferation. The AKT kinase pathway activates mTOR which results in cells proliferation. PI3K induce angiogenesis by activating of hypoxia-inducible factor-1a. Activation of AKT2 promotes GLUT4 translocation leading to the activation of glycogen synthase [31, 41, 42]. Moreover in cancer cells it was shown that GF-1R undergoes nuclear import and both alpha and beta subunits traffic to the nucleus by clathrin-mediated endocytosis. Ligand activated nuclear IGF-1R is phosphorylated and undergoes interaction with chromatin and regulate transcription. This nuclear IGF-1R accumulation is associated with poor prognosis in RCC [43].

### Molecular deregulation of IGF1R pathway and cancerogenesis

IGF-1/insulin pathways were show as significant in cancer research. First of all IGF-1 and insulin share overlapping downstream pathways of cancer cell metabolism. Chronic hyperinsulinemia and diabetes mellitus type 2 were associated with tumor development through the obesity-cancer association [31, 44]. According to the *Werner* et all. IGF1R activation is pre-requisite for malignant transformation. As oncogenic transformation is initiated, cell survival of transformed cells is strongly dependent on IGF-1 signaling [44]. Further multiple studies confirmed the role of IGF −1 and its receptors on RCC cancerogenesis [41, 45]. Deregulated IGF1R kinase activity and its overexpression was reported in multiple cancers including RCC [46–48]. In particular constitutively active IGF1R leads to salivary and mammary adenocarcinomas in transgenic mice [49]. Transgenic overexpression of IGF1R increases epithelial mammary gland hyperplasia and tumor formation [50]. In the RCC cell lines including Caki-2 (from a primary tumor) and SK-RC-52 (from a metastatic tumor) IGF-1 was shown to enhance transforming growth factor-β (TGF-β) signaling and via TGF-β raise IGF-binding protein 3 (IGFBP-3) levels with growth-promoting effect [51].

**Fig. 2:**
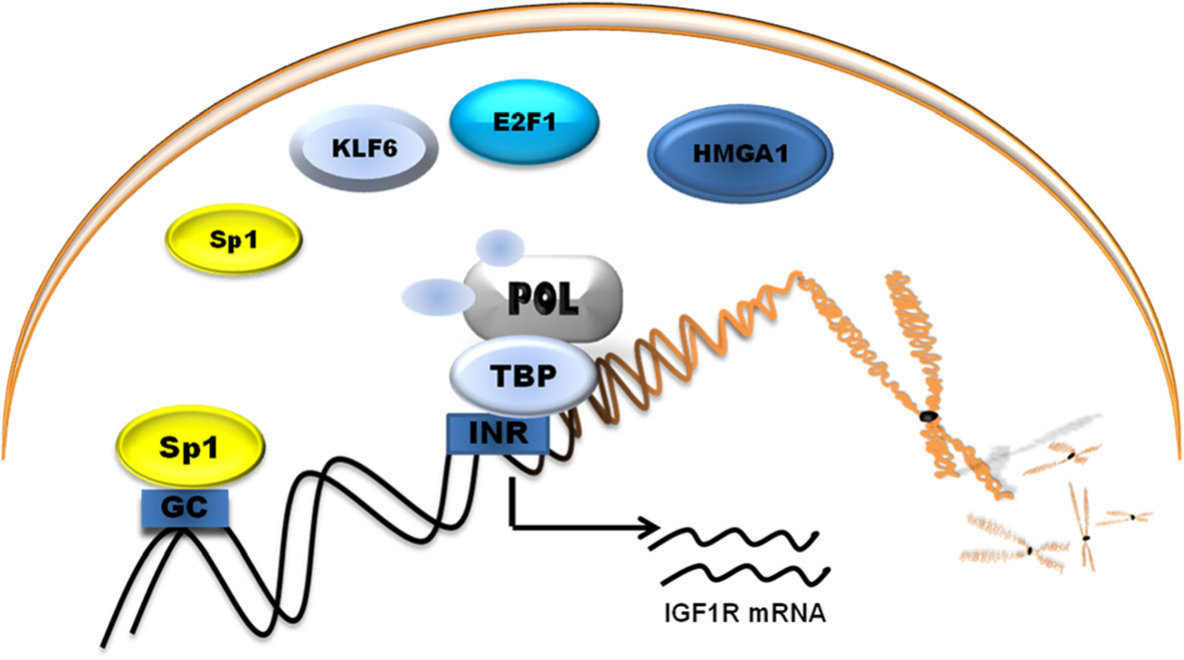
Schematic representation of promoter region of IGF1R and its main transcription factors. Sp1, Specificity protein 1; HMGA1, High mobility group A1; KLF6, Krüppel-like factor 6; E2F1, E2F family of transcription factors; POL, RNA polymerase II; TBP, TATA-binding protein; GC, GC boxes; INR, initiator element

Unlike in most genes, promoter sequence lacks TATA and CCAAT boxes that are usually required for efficient transcription initiation. Instead of TATA box, promoter is highly rich in GC base pairs. The initiator (INR) is located approximately 1000 bp upstream of the coding region. Specificity protein 1 (Sp1) is a key transcription activator of the *IGF1R* gene that binds with high affinity to GC boxes in promoter. The products of tumor suppressor genes like *BRCA1*, *p53*, *VHL* acts through the Sp1 protein [52]. The schematic representation of promoter and other transcription factor shows Fig. 2.

IGF1R activity deregulation is result of loss of tumor suppressor genes or the action of oncogenes [53]. The examples are *p53, BRCA1, WT1* genes. *p53* is the one of the most frequently mutated tumor suppressor and is well known gene associated with cancerogenesis. Product of *p53* gene accumulates in response of DNA demage and results in arrest of cell cycle at G1 phase. Cell can repair DNA or activate apoptotic program. Wild-type p53 supperess the activity of *IGF1R* promoter whereas mutated p53 enhence promoter activity and accelerate tumor growth [54]. Next antioncogene *BRCA1* is linked to the etiology of hereditary of familial breast and ovarian cancer. Wild-type BRCA1 reduce the promoter activity of *IGF1R* whereas mutated BRCA1 lead to enhance *IGF1R* promoter activity and expression of protein. *BRCA1* and *BRCA2* carries have an increased level of IGF1R compared with those with sporadic cancers [55, 56]. *WT1* gene which product is a zinc-finger transcription factor is associated with the etiology of *Wilms tumor* a kind of pediatric kidney cancer [57]. All of these genes products are able to suppress the activity of *IGF1R* promoter, as well as the endogenous levels of IGF1R mRNA [57–59]. IGF1R expression level is raised in non-*BRCA1*-mutated ovarian cancer cells compared with normal tissue and additionally IGF1R levels are significantly increased in *BRCA1*-inactivation ovarian cancer [59]. Moreover investigation in SNP in *IGF-1* and *IGFR-1* indicate that IGF1-pathway polymorphisms are potential prognostic molecular markers in colorectal cancer and pancreatic cancer. There are significantly associated with progression-free survival and/or overall survival (OS) in these cancers [60, 61]. SNP polymorphisms and mutation of *IGF1R* that are associated with cancer represents Table 2.

**Table 2:** SNP’s and mutations of *IGF1R* gene associated with cancers

SNP/mutation	Nucleotide change	Type of cancer	Fuctional feature	Ref.
rs8038415	TT	Breast cancer	*BRCA1* carriers with homozigosity TT at this SNP site experience a 40 % higher risk of breast cancer.	[103]
rs2272037	T > C	Colorectal cancer; Glioma	Significantly related with shorter OS in patients with metastatic colorectal cancer (mCRC). CT and TT associated with increased risk for glioma.	[60] [104]
rs2016347	G > T	Colorectal cancer, glioma	Related to reduced responsiveness to cetuximab treatment. Shorter OS in patients with mCRC. G allele associated with increased risk for Glioma (3’UTR in 3129 site)	[104, 105]
rs8038415	C/T	Non–small cell lung cancer (NSCLC)	Homozygous TT in this SNP had a significantly better OS compared with heterozygous individuals and a trend toward improved survival compared with patients that were homozygous for CC .	[106]
CNV in *IGF1R* gene		Non–small-cell lung cancers (NSCLC)	High *IGF1R* gene copy number harbors positive prognostic value in NSCLC	[107]
Amplification in 15q26		High grade glioma	Unkown	[108]
Amplification in 15q25-26		Alveolar Rhabdomyosarcoma	Related with the rearrangement of *PAX7* gene	[109]
A1374V		Lung squamous cell carcinoma	Unknown; mutation occur in the C-terminal lobe of the kinase catalytic domain	[110]
Deletion S1278		Renal clear cell carcinoma	Unknown; deletion occur in the C-terminal tail region of the receptor	[110]
M1255I		Lung adenocarcinoma	Unknown; mutation occur in in the C-terminal lobe of the kinase catalytic domain	[110]
G596V		Thymic carcinoma	Exonic, Missense	[111]
rs61740868	C/T	Unknown	Unfavorable substitution Arg1216Cys; showed an increase in energy (less favorable change) in comparison with the native structure.	[112]
rs45437300	A/T	Unknown	Nonsense mutation	[112]
rs2229765	A/G	Unknown	Affect splicing regulation; to be associated with higher plasma concentrations of circulating IGF1R	[112]
rs55895813; rs36108138; rs45495500	A/G; A/C; C/T	Unknown	Splicing site	[112]

**Fig. 3:**
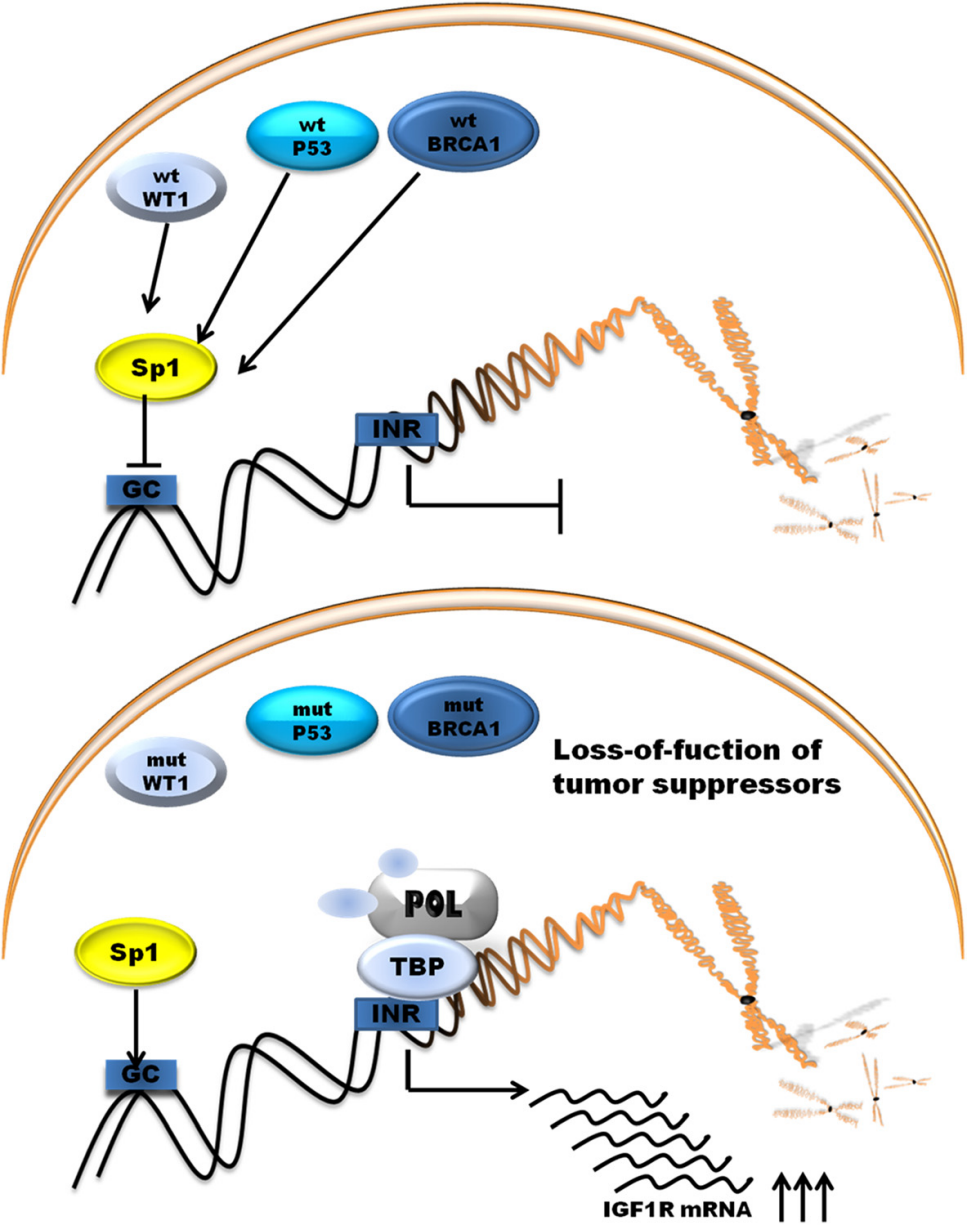
Regulation of promoter activity of IGF1R gene by tumor suppressor genes. POL, RNA polymerase II; TBP, TATA-binding protein; GC, GC boxes; INR, initiator element

The mechanism of action of P53 and BRCA1 involves interaction with Sp1 to repress the expression of *IGF1R* [52]. BRCA1 can inhibit *IGF1R* expression regardless of p53 expression level but not in mutant state of *p53*. WT1 protein does not involve direct interaction with promoter DNA sequence [62]. *Loss-of-function* of that genes leads to up-regulation of *IGF1R* gene. Other tumor suppressor gene *VHL, Von Hippel–Lindau* tumor suppressor also inhibits *IGF1R* promoter activity through interaction with Sp1 protein. In RCC inactivation of *VHL* is associated with *IGF1R* up-regulation [63]. Schematic representation of main tumor suppressor protein acting on promoter is shown on Fig. 3.

### Expression of IGF-1 and IGF1R in kidney

Locally synthesize IGF-1 in kidney acts as a paracrine or autocrine factors. Level of IGF-1 in venous renal blood is higher than in renal arterial blood. There are no strong proves on IGF-1 epithelial expression. Studies indicates connective tissue adjacent to epithelial renal cells to be responsible for local synthesis of IGF-1 [64, 65]. Research on human fetal kidney shows no expression of mRNA of IGF-1 in nephrogenic zone but only probably sequestration of IGF-1 peptide in proximal and distal tubules. Studies on rats and mouse shows the IGF-1 (mRNA and peptide) expression (immunolabeling) in collecting ducts. In situ hybridization studies indicate the mRNA expression in medullary thick ascending limb of henle’s loop [64, 66, 67]. IGF- 1 mediated decline in renal vascular resistance, leads to elevated glomerular perfusion, sodium and water resorption leading in turn to soft tissue swelling and glomerular hypertrophy [65, 68]. *IGF1R* gene expression were detected in whole nephogenic zone including the strong expression in glomeruli and the tubular epithelium of medulla with the least expression in proximal tubules [69]. In contrary Kamenicky et al. shows the strong expression of mRNA of *IGF1R* in proximal tubule obtained from murine cells by microdissection [65, 70].

### Expression of IGF-1 and IGF1R in ccRCC

The type 1 insulin-like growth factor receptor (IGF1R) has an influence on renal cells malignant transformation by induction of cell proliferation, dedifferentiation and anti-apoptotic effect [71, 72]. IGF-1 and IGF1R expression is modulated in kidney development. In mouse model of kidney development IGF-I mRNA is expressed in all cell types with peak in the proximal tubules, peritubular capillaries of the outer medulla and inner cortex one week after birth. The expression of IGF1R in normal proximal tubules was similar to IGF-1 during kidney development until birth [73], but it is poor prognosis factor in RCC [74]. Overexpression of IGF family members was reported observed in oxidative stress (ferric nitrilotriacetate) induced RCC in rats [75]. What is interesting RCC cells with high expression of IGF1R are more resistant to chemotherapy than cells with low expression of that receptor [2]. IGF-I pretreatment levels in RCC patients was predictive to impaired response to interleukin-2 (IL-2) therapy [76]. In SN12K1 cells (cell line derived from metastatic RCC) it was shown that RCC express IGF-I and IGFBP-3, and autocrine IGF-I and IGFBP-3 stimulate and inhibit growth respectively. These cells are also are responsive to exogenous IGF-I stimulation - DNA synthesis is increased. These RCC cells are also responsive to exogenous IGF-I stimulation [77]. Further experiments on RCC cell lines - Caki-2 (primary tumor) and SK-RC-52 (metastatic tumor) – have shown that IGF-I enhances transforming growth factor (TGF)-beta signaling including phosphorylation and nuclear translocation of mothers against decapentaplegic homolog 2 (Smad2). In turn TGF-beta promotes IGFBP-3 production [51]. In mice model injection of MZ-4–71 - growth hormone-releasing hormone antagonist - reduced the IGF-1 induced growth of Caki 1 RCC cell line derived tumors [78].

In first clinical reports high serum IGF-I levels have been associated with an increased risk of developing RCC [77]. At the same time in the analysis of 256 patients serum IGF-1 was not correlated with tumor stage or grade, but was independent favorable prognostic factors in a multivariable analysis [79]. More recently the prospective study of 29 133 Finnish male smokers reported that men with IGF-I levels >113 ng ml(−1) were 59 % less likely to develop RCC than men with levels below or =113 ng ml(−1). Among those 100 men with RCC the IGF binding protein-3 (IGFBP-3) levels did not alter the association [80]. In the study of 90 patients with ccRCC and 20 normal renal tissue samples, hyper-methylation of IGFBP-3 was not found, but in 786–0, ACHN, HRC51 and HRC59 cell lines methylation of *IGFBP-3* was observed [81]. Moreover IGF-I and its binding proteins IGFBP-3 and −6 are up-regulated in ccRCC tumor tissues [77]. IGF1R expression was also associated with ccRCC and indicated molecular prognostic marker and potential targets for therapeutic intervention. Data of 280 patients who had ccRCC treated with radical nephrectomy showed that IGF1R expression had a 70 % increased risk of death than patients who had tumors without IGF1R expression [82]. Finally nuclear IGF-1R was detected in primary renal cancer tissues of high proliferation rate and was associated with adverse prognosis [43].

### Pathological and clinical grading system and IGF1R status

Fuhrman nuclear grading system correlates with staging of ccRCC. Studies on expression of IGF1R and Furman score in 68 ccRCC showed the statistically significance increasing correlation between Fuhrman grading and IGF1R staining [83]. Also the high serum concentration of IGF1R is associated with the risk of many cancers such as breast, prostate, colorectal, and lung cancers [71]. Overexpression of IGF1R is related with poor prognosis in many human cancers besides the renal cancer also in breast and ovarian cancers [84, 85]. Antisense strategies against the IGF1R suppresses the expression of IGF1R can abolish cell transformation [71, 83]. No correlation was observed between serum IGFBP-3 levels and RCC [79], but high expression of IGFBP-3 was found in ccRCC tumors. Fuhrman grades 3 and 4 ccRCC tumors showed higher IGFBP-3 expression than low grade tumors [80, 86].

Although the IGF1R and Insulin receptor (IR) show great homology and interaction they have different relation to the prognosis in ccRCC. Lkhagvadrj et al. investigated the role of IR expression in 126 ccRCC cases. Researchers showed that there was no differences of total IR protein between tumor and non-tumor but immunoreactivity of IR in tumor was mostly observed in nuclear but in non-tumor tissue in nuclear and cytoplasm. The expression of IR was elevated in low-grade tumor but not in higher-grade. Also the IR expression was inversely correlate with Furman nuclear grade and TNM, pathologic T stage in ccRCC. Higher expression of IR correlated with cystic changes in RCC which is favorable prognostic factor. What is interesting IR expression was not related to the diabetes presence. The authors suggest that although IR and IGF1R share the major down-stream signaling pathway, in RCC there are specific substrates for each receptor [87].

### Interaction of VHL and IGF1R

Hereditary RCC is commonly associated loss or mutation of the *Von Hippel-Lindau* (*VHL*) tumor suppressor gene. *VHL* mutation was first discovered in patients with VHL disease by Latif F et al. [88] and *VHL* mutation or loss is identified in 60-90 % of sporadic RCC cases [7, 89]. Next 20 % of ccRCC show silencing of *VHL* expression by methylation in promoter region [90]. VHL gene encodes protein with ubiquitin ligase E3 activity directing hypoxia-inducible factor-1α (HIF-1α) for degradation [87]. HIF-1α regulate hypoxia response and promote angiogenesis, cell migration, and metabolism [7, 91] via VEGF, PDGF and TGF [92]. Independently of oxygen status IGF1R activates HIF-1α protein by suppressing VHL and promote RCC development [63]. At the same time IGF-1-mediated signaling is inhibited in the presence of wild-type VHL and VHL decrease stability of IGF-1R [93]. In RCC cells beta-domain of VHL interact with protein kinase C delta (PKCD) and inhibits its association with IGF-IR and subsequent downstream signaling [94]. It was also shown that receptor for activated C kinase 1 (RACK1) serves as a direct mediator between loss of pVHL function and IGF-IR signaling in RCC cells. Upon IGF-1 stimulation, pVHL-deficient RCC cells exhibit high rate of RACK1/IGF-IR binding and up-regulated IGF-1R tyrosine kinase activity, phosphoinositide 3-kinase/serine-threonine kinase Akt (PI3K/Akt) signaling and matrix metalloproteinase-2 (MMP-2) activity and high cellular invasiveness [95]. VHL protein has no influence on ubiquitination of IGF1R. On the contrary the activity of *IGF1R* expression is regulated by VHL at the transcriptional level and is mediated by Sp1 transcription factor. Sp1 protein is sequestered by VHL and act on promoter of *IGF1R*. Loss of *VHL* gene increases therefore IGF1R mRNA stability. The levels of IGF1R is higher in ccRCC samples than in benign renal tumors which could be associated with *VHL* mutation rate in RCC [63, 96]. At the same time depletion of IGF1R enhance the chemosensitivity of ccRCC, but this effect is significant in cells with no functional *VHL.* Depletion of *IGF1R* changed sensitivity to mTOR inhibitors, 5-FU, etoposide but not cisplatin [2].

## Conclusions

The role of IGF-1/Insulin pathway in cancerogenesis remain unclear. IGF-1 and insulin share major downstream regulation pathway and both are engaged in cancerogenesis and diabetes [97]. There are evidences that diabetic patients have risk of development of renal cancer. Increase in mortality and incidence of renal cell carcinoma among diabetic patients is linked to hyperinsulinemia and obesity. The interaction of hyperglycemia, hyperinsulinemia causes the insulin resistant state and contribute to central adiposity which result in chronic inflammation. The adipose tissue-derived cytokines like resistin, tumor necrosis factor α (TNF-α) and interleukin 6 (IL-6) promotes persistent inflammation and result in genetic instability, putting cells at risk to malignant transformation. Nuclear factor-kB is strongly activated by TNF-α what is associated with cell proliferation and surviving malignant cells [97]. Associations between hyperinsulinemia and increased circulating levels of IGF-1 is proposed to be involved in carcinogenesis. IR and IGF-1 has a nuclear translocation potential and were postulated to be nonclassical transcription factors. IGF1R physically interact with gDNA and also stimulates itself expression. Study of nuclear IGF1R in primary renal cancer cells revealed that IGF1R expression was associated with poor prognosis in renal cancer [98]. Cell culture experiments have proven that active IGF-IR is necessary for cell transformation by multiple cellular and viral oncogenes. IGF-IR signaling regulate the cell cycle, cell survival/proliferation, cell-cell interactions, ECM attachment, cell motility and metastatic spread. In mice model IGF-IR overexpression promotes tumor growth and metastases development, whereas down-regulation of IGF leads to slower tumor development [3]. In general anti-diabetic treatment may increase the risk of RCC cancer development, but large prospective data is not available at this point of time. Exception is metformin an oral anti-diabetic drug has a RCC-protective effect as it interacts with the IGF signaling pathway which results in inhibition of proliferation and apoptosis regardless the p53 status [99].

## Abbreviations

ccRCC, Clear cell renal cell carcinoma; CTLA-4, cytotoxic T lymphocyte antigen 4; IGF-1, Insulin-like growth factor-1; IGF1R, IGF-1 receptor; IGFBPs, IGF-binding proteins; PD-1, programmed cell death 1; PDGFR, Platelet-derived growth factor receptors; RCC, renal cell carcinoma; VEGF, Vascular endothelial growth factor; VHL, Von Hippel–Lindau protein
